# Corticostriatal Plastic Changes in Experimental L-DOPA-Induced Dyskinesia

**DOI:** 10.1155/2012/358176

**Published:** 2012-05-13

**Authors:** Veronica Ghiglieri, Vincenza Bagetta, Valentina Pendolino, Barbara Picconi, Paolo Calabresi

**Affiliations:** ^1^Laboratorio di Neurofisiologia, Fondazione Santa Lucia, IRCCS, Via del Fosso di Fiorano 64, 00143 Rome, Italy; ^2^Clinica Neurologica, Dipartimento Specialità Medico Chirurgiche e Sanità Pubblica, Università di Perugia, S. Maria della Misericordia, 06156 Perugia, Italy

## Abstract

In Parkinson's disease (PD), alteration of dopamine- (DA-) dependent striatal functions and pulsatile stimulation of DA receptors caused by the discontinuous administration of levodopa (L-DOPA) lead to a complex cascade of events affecting the postsynaptic striatal neurons that might account for the appearance of L-DOPA-induced dyskinesia (LID). Experimental models of LID have been widely used and extensively characterized in rodents and electrophysiological studies provided remarkable insights into the inner mechanisms underlying L-DOPA-induced corticostriatal plastic changes. Here we provide an overview of recent findings that represent a further step into the comprehension of mechanisms underlying maladaptive changes of basal ganglia functions in response to L-DOPA and associated to development of LID.

## 1. Introduction

In Parkinson's disease (PD), degeneration of dopaminergic neurons of the *substantia nigra* causes critical reduction in dopamine (DA) levels in the target areas. The subsequent abnormal DA receptor stimulation exerts its main effects in the striatum, the principal input structure of basal ganglia-thalamo-cortical network, producing changes in input integration that lead to imbalance between direct and indirect striatofugal pathways and dysfunctional changes in basal ganglia output.

Impairment in the induction of the two forms of striatal synaptic plasticity, the long-term depression (LTD) and the long-term potentiation (LTP), has been found to correlate with DA depletion and onset of symptoms in experimental models of PD. DA depletion initially affects LTP and then, when symptoms are fully manifested, also LTD is impaired [1].

The resulting motor symptoms are effectively treated with a replacement therapy that uses the DA precursor L-3,4-dihydroxyphenylalanine (L-DOPA) to rescue striatal DA-dependent neuronal activity. However, L-DOPA treatment does not arrest disease progression and, with time, neuronal degeneration advances and leads to the emergence of a complex pattern of alterations that involves other basal ganglia nuclei, causing symptoms that are refractory to conventional therapy. In addition, the initial excellent antiparkinsonian effects of L-DOPA are lost in the long run, and the route of drug administration utilized in the clinical practice leads to a pulsatile stimulation of DA receptors that causes a broader neuronal destabilization. Therefore, new motor complications unavoidably develop, resulting in L-DOPA-induced dyskinesia (LID), a very disabling long-term side effect of L-DOPA therapy associated with the loss of corticostriatal bidirectional plasticity [2].

The expression of an aberrant plasticity following chronic L-DOPA treatment has been also demonstrated in PD patients [3–5], further supporting the notion that a treatment with a drug able to ameliorate disease symptoms can be associated with the recovery of a selective form of synaptic plasticity.

This review provides an overview of papers that contributed to characterize the plastic changes occurring at striatal synapses in experimental models of LID. After a description of the main forms of DA-dependent synaptic plasticity at glutamatergic corticostriatal synapses, we will introduce seminal studies focusing on the plastic changes observed in dyskinetic models. We will then review the most recent papers that further explored mechanisms underlying L-DOPA-induced changes in experimental PD models and discuss recent findings that, in our opinion, represent new promising avenues to future electrophysiological studies on dyskinetic animals.

## 2. DA-Dependent Synaptic Plasticity at Corticostriatal Synapses

At corticostriatal synapses, repetitive cortical activation can induce either LTD or LTP in the striatal medium spiny neurons (MSNs), depending on the level of membrane depolarization, the subtype of glutamate receptor activated [6–8], and the interneuronal subtypes involved in the induction process [9]. Unique characteristic of striatal neurons is that DA critically regulates both the induction and the maintenance of neuroplasticity via DA D_1_-like (D_1_) and D_2_-like (D_2_) receptors activation. Specifically, DA acting on D_1_ receptors cooperates to the induction of LTP, whereas activation of both D_1_ and D_2_ receptors is required for LTD [2, 10, 11].

Electrophysiological studies in corticostriatal slices from 6-hydroxydopamine- (6-OHDA-) lesioned parkinsonian rats have shed light on the pivotal role that DA exerts in modulating glutamatergic transmission and synaptic plasticity within the striatum [12].

A complete DA denervation abolishes both forms of corticostriatal plasticity [11, 13] that can be restored by treatment with either DA receptor agonists or the DA precursor L-DOPA [2, 11, 14].

We have recently shown that distinct degrees of DA denervation influence the two forms of plasticity in different ways, as full DA denervation blocks the induction of both LTP and LTD, while partial DA depletion allows LTP induction but selectively alters its maintenance, leaving LTD induction unaffected [1].

A third form of striatal plasticity, distinct from LTD, called synaptic depotentiation, results from the reversal of an established LTP by the application of a low-frequency stimulation (LFS) of corticostriatal fibers [2, 15]. This form of plasticity critically relies on glutamatergic N-methyl-D-aspartate (NMDA) receptor activation [16] and striatal endogenous tone of acetylcholine [17]. During LTP, protein kinase A (PKA), a downstream effector of DA D_1_ receptors, phosphorylates and activates DA- and cAMP-regulated phosphoprotein of 32 KDa (DARPP-32), a potent inhibitor of protein phosphatase 1 (PP-1). PP-1 dephosphorylates several downstream targets of PKA, thereby amplifying behavioral responses produced by activation of cAMP signalling [18–20], and it is necessary for depotentiation, as this form of plasticity is blocked by application of PP-1 inhibitors.

DA and glutamate receptors functional interaction in the striatum has been shown to regulate locomotion, positive reinforcement, attention, and working memory. In particular, activation of D_1_ receptors is needed for the correct integration of cortical glutamatergic signals to the striatum [21]. In striatal MSNs, D_1_ receptors are located within dendritic spines, where they colocalize with NMDA receptors [22, 23] regulate the rapid trafficking of NMDA receptor subunits [24] and the potentiation of NMDA responses [25], leading to activity-dependent adaptive changes [10] and also to the activation of excitotoxic pathways. Among the signalling cascades regulating D_1_ receptor-dependent enhancement of NMDA responses in the striatum, the most important involves PKA- and DARPP-32-regulated phosphorylation of NMDA receptor NR1 subunits [26].

D_1_ DA receptor stimulation also enhances phosphorylation of the alpha-amino-3-hydroxy-5-methyl-4-isoxazolepropionic acid (AMPA) glutamate receptor subunit GluR1 at the PKA site, increases surface expression of AMPA receptors, and facilitates their synaptic insertion in several brain areas [27, 28].

Besides the concurrent activation of glutamatergic and dopaminergic receptors, activity-dependent plasticity of glutamatergic synapses at MSNs is also modulated by other signalling pathways like endocannabinoids, adenosine (presynaptically), and metabotropic glutamate (pre- and postsynaptic) receptors [29] and by striatal interneurons [30, 31], which represent a minority of total striatal population but play a crucial role in the modulation of basal ganglia function, contributing to the processing of corticostriatal information [9, 13, 32]. In particular, two interneuronal subtypes have been suggested to play a critical role in the pathogenesis of LID: the large-aspiny cholinergic interneurons and the nitric-oxide-synthase- (NOS-) positive interneurons.

The cholinergic interneurons, which represent the main source of acetylcholine within the striatum [33], play a permissive role in corticostriatal synaptic plasticity by modulating the striatal cholinergic tone [9, 34]. These interneurons respond to cortical stimulations with long lasting changes of synaptic efficacy [17, 35] and are important sites of interaction among DA, adenosine, and endocannabinoid receptor signalling systems [36], further supporting the idea that cholinergic interneuronal activity contributes to striatal-dependent learning and motor habit formation.

The NOS-immunoreactive neurons represent, along with the cholinergic interneurons, the other interneuronal subtype that plays an important role in the induction of LTD [13, 31, 34]. These interneurons express mRNA encoding for ionotropic glutamate receptors that appear to be coupled to nitric oxide production [37–40]. Nitric oxide activates soluble guanylyl cyclase (sGC), which in turn induces increases of intracellular cyclic guanosine monophosphate (cGMP) levels to activate the protein kinase G (PKG) [41–43], whose levels are regulated by the action of phosphodiesterases (PDEs), a family of enzymes responsible for the conversion of cGMP to GMP. Accordingly, pharmacological LTD can occur in MSNs following the application of phosphodiesterases inhibitors [13], as a consequence of increased cGMP levels. In fact, the amount of this nucleotide is crucial for the activity of PKG and DARPP-32, which in turn control the phosphorylation of AMPA receptor, a main player in the induction of LTD [10].

In summary, the integrative action exerted by striatal projection neurons on the converging information arising from the cortex, the nigral DA neurons and the striatal interneurons, shapes the activity of neurons throughout the entire basal ganglia circuitry.

## 3. L-DOPA-Induced Plastic Changes at Glutamatergic Synapses

The effects of L-DOPA administration in the DA-depleted striatum have been extensively studied in experimental models of LID, leading to the concept that a combination of presynaptic and postsynaptic maladaptive changes is needed for the parkinsonian animals to develop dyskinesia [3, 44, 45].

During progressive degeneration of nigrostriatal terminals, sprouting of DA terminals and reduced DA uptake contribute to preserve DA striatal levels [46], and increase in glutamate transmission is observed in corticostriatal pathway [47–51] as well as in basal ganglia output nuclei [52, 53].

However, such presynaptic adaptive changes together with changed presynaptic and postsynaptic DA receptor sensitivity and density lead to an altered substrate in which L-DOPA exerts its actions. Thus, initially, L-DOPA is converted into DA, stored in synaptic vesicles, and released by surviving DA-releasing terminals. However, when degeneration advances, DA catabolism and uptake are reduced and decarboxylation of L-DOPA to DA and release occur in non-dopaminergic cells [54, 55], causing a failure in the buffering of DA levels.

The consequent large fluctuations in extracellular DA concentrations, mainly relying on the drug-dosing cycle, contribute to the establishment of further morphological and functional changes at both pre- and postsynaptic levels.

During chronic treatment with L-DOPA, several postsynaptic pathways downstream DA and glutamate receptors activation are progressively dysregulated, causing a loss of control of phosphorylation cascades with increase of phosphorylated striatal substrates such as NMDA receptor subunits [56, 57], AMPA receptor subunits [58], and extracellular signal-regulated kinase (ERK)1/2 [44, 58–60]. One crucial pathway that has been extensively investigated is the signalling activated by D_1_ receptor stimulation [61]. In the DA-depleted striatum, in fact, chronic L-DOPA treatment, through stimulation of sensitized D_1_ receptors causes hyperactivation of PKA and increased striatal phosphorylation of DARPP-32 at the threonine-34 residue [58, 62]. As above mentioned, this protein plays a pivotal role in the synaptic alterations caused by unphysiological stimulation of DA D_1_ receptors. In fact, DARPP-32 is a potent inhibitor of PP-1 activity, which in turn is necessary to depotentiate the synapse.

A critical link between abnormal involuntary movements (AIMs), resembling human dyskinesia, and loss of bidirectional synaptic plasticity at corticostriatal synapses of dyskinetic rats has been firstly provided by our group [2, 63]. In the unilateral 6-OHDA model of PD, chronic treatment with either high or low doses of L-DOPA is able to restore LTP expression. However, in a consistent number of treated animals, the corticostriatal glutamatergic signalling undergoes further adaptive changes and AIMs develop [2, 64, 65]. Hyperphosphorylation of DARPP-32 at the threonine-34 residue occurs selectively in animals developing dyskinetic behavior and is associated to the loss of capability to depotentiate the corticostriatal synapse [2]. Moreover, in dyskinetic animals, prolonged L-DOPA treatment remarkably reduces synaptic D_1_/NMDA receptor complexes without changing their interaction [23]. However, further complex molecular alterations take place at glutamatergic synapse that are strictly correlated to abnormal synaptic plasticity and motor behavior in L-DOPA-treated dyskinetic rats [2, 16]. Specifically, levels of NR2A subunit are higher in dyskinetic animals compared to nondyskinetic ones, and this effect is paralleled by decreased levels of NR_2B_ subunit, which are found increased in extrasynaptic sites [16]. Such redistribution of NMDA receptor subunits is associated with alterations in the binding of NMDA receptor subunits with their cargo proteins, in particular, SAP-97 and SAP-102 [16]. Impairment of the physiological trafficking of NMDA receptor subunits from the reticulum toward the postsynaptic density may, therefore, determine the enhancement of NMDA receptor signalling in dyskinesia.

Accordingly, pharmacological manipulation aimed at reducing synaptic localization of NR_2B_, and consequently increasing NR_2A_/NR_2B_ ratio at synaptic sites, causes in nondyskinetic subjects a worsening of motor symptoms with appearance of dyskinetic behaviours [16]. Intracerebral administration of a cell-permeable peptide (TAT2B), able to alter the NR_2B_ synaptic localization by perturbing its binding with scaffolding proteins, causes loss of depotentiation that correlated with AIMs in nondyskinetic animals [16].

Taken together, these findings support the notion that abnormal activation of PKA and concomitant hyperphosphorylation of DARPP-32 observed in experimental models of LID are two of the main causes of changes in the state of phosphorylation state of target effector proteins, with consequent profound repercussion on the excitability and plasticity of striatal MSNs.

## 4. Novel Insights into L-DOPA-Induced Changes in Corticostriatal Synaptic Plasticity

Three new studies have investigated further on the mechanisms underlying the loss of synaptic scaling down at corticostriatal synapses.

Gardoni and coworkers have recently shown that pharmacological manipulations interfering with the interactions between NMDA receptor subunits and their scaffolding proteins, responsible for their trafficking and correct assembly at synaptic membranes, prevents the unbalance of NR_2A_/NR_2B_ subunit ratio by reducing the synaptic localization of NR_2A_ subunit. Systemic coadministration of the cell-permeable peptide TAT2A and L-DOPA reduces the percentage of animals developing dyskinesia [66]. However, once the AIMs are established, the administration of TAT2A fails to reduce incidence of dyskinesia, indicating that altered NMDA receptor composition has a critical role in initiating the dyskinetic phenotype. Moreover, these data support the concept that molecular disturbances of the glutamatergic synapse, initially caused by DA denervation, create a pathological substrate that induce and maintain the overworking synapse at an altered steady state that triggers the development of LID [2, 16].

A further advance in the characterization of bidirectional synaptic plasticity following L-DOPA therapy has been made in a recent study conducted by our group. Based on the evidence that striatal cGMP signalling is decreased in dyskinetic animals [67], we explored the possibility that LTD, which strictly relies on the nitric oxide-dependent activation of PKG, was altered following L-DOPA treatment. We found that MSNs recorded from L-DOPA-treated dyskinetic parkinsonian rats do not express activity-dependent LTD. Increase of cGMP levels by PDEs inhibitors leads to the activation of PKG, mimicking the action of nitric oxide released from NOS-positive neurons that represents a critical factor for LTD induction following HFS [13]. Accordingly, application of a low dose of PDEs inhibitor, unable to induce *per se* a pharmacological LTD in dyskinetic parkinsonian rats, is sufficient to rescue activity-dependent LTD in these animals.

Interestingly, application of PDEs inhibitors induces pharmacological LTD in both dyskinetic and nondyskinetic rats but not in untreated parkinsonian animals, indicating that the presence of endogenous striatal DA represents a critical condition also for the induction of this form of pharmacological plasticity. Local injection of these drugs into the striatum of dyskinetic rats rescues LTD and reduces the dyskinetic response to L-DOPA [62].

This phenomenon, together with the loss of depotentiation [2], is in line with the view that LID is caused by impaired control of striatal excitatory synapses with excessive increase of glutamatergic transmission.

Accordingly, the third study by Usiello and coworkers investigated the contribution of a basal hyperglutamatergic tone in the development of dyskinesia associated to altered DA-dependent bidirectional synaptic plasticity.

Using mutant mice lacking the D-Aspartate Oxidase (Ddo) enzyme (Ddo^−/−^ mice), showing nonphysiological high levels of the excitatory free D-amino acids D-aspartate and NMDA [68], they found that a condition of persistent hyperstimulation of glutamatergic transmission results in an aberrant striatal synaptic plasticity. In the MSNs recorded from Ddo^−/−^ mice, similar to what observed in dyskinetic animals, LFS protocol fails to reverse the synaptic transmission levels to those preceding LTP.

When subjected to 6-OHDA lesion, Ddo^−/−^ mice display increased sensitivity to L-DOPA and early onset of dyskinetic behavior [69] further supporting the concept that increased glutamatergic release is a critical risk factor to develop LID.

## 5. New Promising Avenues to Further Investigate L-DOPA-Induced Corticostriatal Plastic Changes

In the recent past, new molecular targets for LID have been explored that may play a critical role in the synaptic alterations underlying plastic changes in the DA-depleted striatum exposed to long-term L-DOPA. An important contribution to the understanding of mechanisms involved in the development of dyskinesia has been provided by the evidence that not only ERK but also its downstream targets, including molecules involved in the regulation of protein translation and gene transcription [60, 70], are entailed in the dysregulation of phosphorylation cascades induced by L-DOPA. The group of Fisone and coworkers has recently demonstrated that abnormal activation of ERK is associated to increased signalling of mammalian target of rapamycin complex 1 (mTORC1) via inhibitory control of tuberous sclerosis complex (TSC) 1 and 2 that, in turn, suppresses activation of Ras homolog enriched in brain (Rheb), a highly conserved member of the Ras superfamily of G-proteins, ultimately responsible for mTORC1 activity. Coadministration of L-DOPA and rapamycin, a selective allosteric inhibitor of mTOR complex, diminishes the development of LID without interfering with the therapeutic effects of L-DOPA [56]. Recently, it has been shown that besides Rheb, another small G protein, the Ras homologue enriched in striatum (Rhes), is critically involved in the pathological upregulation of mTORC1 during LID [71]. These data further strengthen the hypothesis of an involvement of mTORC1 signalling in LID, as Rhes knockout mice show reduced dyskinesia in response to L-DOPA, but the therapeutic improvement of limb motion remains unchanged. Interestingly, a role of mTORC1 in synaptic plasticity has been recently put forward [72]. Relevant to corticostriatal pathway, it has been shown that inhibition of mTORC complexes is able to block a pathological form of persistent LTP associated to increased glutamatergic signalling and neurodegeneration [73].

Taken together, these data suggest that enhanced mRNA translation, leading to abnormal local protein synthesis in the cytoplasm, may participate in the development of aberrant enhancement of synaptic strength, as observed in LID.

Another intriguing aspect that has been recently investigated is the capability of L-DOPA to exert its action through nondopaminergic systems. Indeed, as PD progresses, degeneration of nigrostriatal terminals also advances, and L-DOPA is converted in DA, stored, and released also from other cellular elements within the striatum, including the serotonin (5-HT) terminals [54, 74, 75]. This action might have both beneficial and detrimental consequences in that it allows L-DOPA to maintain DA levels in the virtual absence of dopaminergic neurons but it also enhances the non-physiological DA receptor stimulation as the feedback control of DA release is absent in the 5-HT system. This might have important implications for corticostriatal synaptic plasticity as unregulated DA transmission may induce further adaptive rearrangement of DA/glutamatergic ionotropic receptors interactions at postsynaptic sites that would critically affect the bidirectional synaptic plasticity.

The hypothesis of the involvement of 5-HT terminals in LID has gained support from recent evidence showing that lesion of the 5-HT system by 5,7-dihydroxytryptamine [75] or pharmacological manipulation of serotoninergic transmission [54, 74] significantly reduces L-DOPA-induced increase of extracellular DA levels in the striatum and abolishes dyskinetic movements in parkinsonian rats chronically treated with L-DOPA [54]. However, decrease of corticostriatal glutamate release could be another mechanism underlying additional antidyskinetic effect [76–78].

A potent synergistic interaction between 5-HT_1A_ and 5-HT_1B_ receptors in counteracting the induction of dyskinetic movements has also been demonstrated in the 1-methyl-4-phenyl 1,2,3,6-tetrahydropyridine- (MPTP-) treated macaques, in which administration of 5-HT_1A_ and 5-HT_1B_ agonists reduces the upregulated levels of FosB, the main postsynaptic striatal marker for LID [79, 80].

Most recently, it has been demonstrated that profound structural changes are associated to the capability of serotoninergic terminals to release DA as “false transmitter.” Cenci and coworkers provided evidence that L-DOPA treatment induces the sprouting of 5-HT axon terminals (increased number of synaptic contacts between 5-HT terminals and striatal neurons) [55]. This specific morphological feature positively correlates with the severity of dyskinesia as shown by increased binding levels of the plasma membrane 5-HT transporter in both experimental models (rodents and nonhuman primates) and in PD patients subjected to L-DOPA therapy. Such increase was correlated with the dyskinetic score and paralleled by the upregulation of brain-derived neurotrophic factor (BDNF) expression [55, 81], which exerts complex functional and structural actions within the striatum.

These results are consistent with the evidence that increased concentrations of striatal BDNF are associated with LID [82] although the role of this neurotrophin in LID development is still under debate [83].

A link between BDNF and LID is also suggested by the fact that striatal BDNF is regulated by the activity of another nondopaminergic pathway involved in the development of LID, the striatal purinergic system. Indeed striatal adenosine, through A_2A_ receptors, has been suggested to play a pivotal role in the regulation of BDNF function and levels in the brain [84, 85] and it has been also implicated in the development of LID [86].

Presynaptically, A_2A_ receptors act to finely tune glutamate release from corticostriatal terminals and they are also present postsynaptically on striatopallidal MSNs of the indirect pathway that express DA D_2_ receptors.

In control condition, concomitant activation of DA D_2_ receptors and blockade of A_2A_ adenosine receptors is able to decrease striatal glutamatergic transmission [87]. This interaction is made possible by a retrograde action of endocannabinoids released by postsynaptic MSNs and acting on CB1 cannabinoid receptors located on glutamatergic terminals [36] suggesting that the convergence of DA D_2_ and A_2A_ signalling systems on the endocannabinoids pathway represents a potent feedback mechanism to control glutamatergic transmission in the striatum. While in control condition, concurrent activation of D_2_ and blockade of A_2A_ are necessary to reduce glutamate release via an endocannabinoid-dependent mechanism, in DA-depleted animals, D_2_ receptor agonism alone is able to reduce glutamatergic transmission due to D_2_ receptor sensitization. This effect could be further enhanced by A_2A_ receptor antagonists providing a solid experimental support for the combined use of D_2_ receptor agonists and A_2A_ receptor antagonists in clinical settings. In fact, alterations in A_2A_ receptor expression and signalling have been extensively observed in PD patients undergoing L-DOPA therapy and in experimental models of LID and A_2A_ antagonists have proven to be effective in clinical and preclinical studies [86].

Notably, striatal cholinergic interneurons, coexpressing D_2_ and A_2A_ receptors, are also interested in this pharmacological modulation, since concomitant activation of D_2_ DA receptors and blockade of A_2A_ receptors reduces the firing rate of this neuronal subtype and muscarinic M_1_ receptor antagonism blocks the D_2_/A_2A_ receptor-mediated modulation of excitatory transmission in both D_2_- and D_1_-expressing MSNs [36]. These results are in agreement with previous studies showing altered acetylcholine signalling in DA-denervated striatum [88] resulting in a loss of feedback control of acetylcholine release [89]. Striatal acetylcholine levels critically determine the direction of synaptic plasticity at corticostriatal synapses with low levels of acetylcholine facilitating LTD and high levels facilitating LTP [90].

Taken together, these data suggest a strong involvement of the striatal cholinergic interneurons in LID pathogenesis. A recent paper [91] shows that in animals lacking the transcription factor Pitx3, modeling PD, chronic L-DOPA enhances baseline and DA-induced firing rate in striatal cholinergic interneurons. This effect is seen also in 6-OHDA-lesioned mice and is associated with increased phospho-ERK immunoreactivity in this neuronal population as inhibition of ERK is able to restore firing rate at control values [91]. In both the unilateral lesion and the genetic models, chronic L-DOPA caused development of LID that was attenuated by administration of dicyclomine, a muscarinic antagonist, without affecting L-DOPA's beneficial antiparkinsonian action.

**Figure 1:**
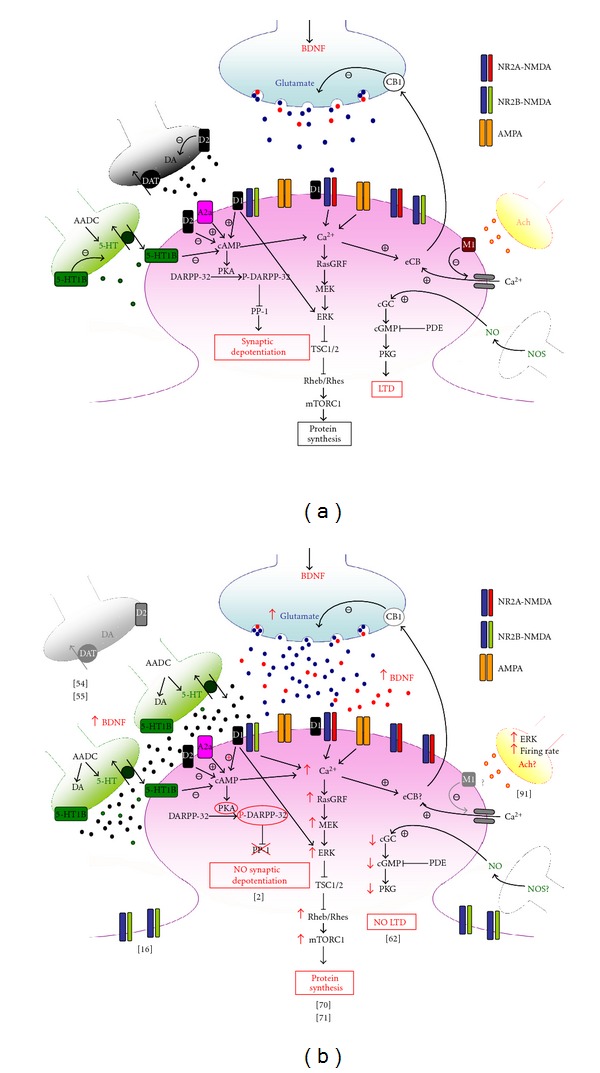
(a) In control condition, dopamine (DA) transmission is regulated by feedback control of release from nigrostriatal terminals (black) through D2 autoreceptors and uptake processes. DA binds striatal postsynaptic D1 receptors inducing the formation of cAMP, which in turn favours the activation of PKA, able to phosphorylate and activate DA- and cAMP-regulated phosphoprotein of 32 KDa (DARPP-32) and extracellular signal-regulated kinase (ERK). Once phosphorylated, DARPP-32 is able to inhibit protein phosphatase 1 (PP-1). Glutamate (blue) and BDNF (red) are released from corticostriatal terminals into the striatum. Glutamate release is regulated by endocannabinoids (eCB) activated by increases in intracellular calcium (Ca^2+^) concentrations through adenosine A2a and muscarinic M1 receptors activation, among other mechanisms, and retrogradely released by postsynaptic striatal neurons. Once released, glutamate activates metabotropic as well as NMDA and AMPA ionotropic receptors, whose activity and surface expression at postsynaptic membrane is also regulated by D1 receptors. In serotoninergic afferents, 5-hydroxytryptophan is converted to serotonin (5-HT) (green) by Aromatic-L-Amino Acid Decarboxylase (AADC) and released into the striatum. Cholinergic and nitric oxide synthase (NOS)-positive interneurons cooperate to induction of corticostriatal LTP and LTD. (b) In dyskinetic state L-DOPA is converted to DA by AADC and released from serotoninergic terminals in unregulated manner. Higher levels of striatal BDNF may support morphological changes in serotoninergic neurons. Excess of DA abnormally stimulates D1 pathway with hyperphosphorylation of ERK and uncontrolled activation of PKA that results in hyperphosphorylation of DARPP32, which persistently blocks PP-1 causing loss of synaptic depotentiation. Abnormal D1 receptor stimulation is associated to increased intracellular Ca^2+^ levels and dysregulation of NMDAR subunit composition with reduction of NR2B-containing NMDAR at synaptic sites, leading to increase in NR2A/NR2B ratio that has been suggested to have a role in the loss of depotentiation. Hyperactivation of ERK through convergent altered signalling pathways brings to increased inhibition of tuberous sclerosis complex (TSC)1/2, and consequent disinhibition of Rheb/Rhes, leading to excessive increase of signalling of mTORC1 that, in turn, exerts its long term effects through changes in protein synthesis. After chronic L-DOPA, cholinergic interneurons show increased phospho-ERK immunoreactivity and higher firing rates with increased release of acetylcholine (Ach). Striatal cGMP signalling is decreased and corticostriatal LTD, which strictly relies on the nitric-oxide- (NO-) dependent activation of protein kinase G (PKG) is abolished in dyskinetic state.

These findings provide new lines of evidence that L-DOPA exerts its widespread action at multiple levels in the functional organization of the striatum (Figure 1). However, a clear-cut definition of a scenario comprising the various maladaptive changes is made difficult by the fact that striatal response to DA-denervation and subsequent DA replacement may vary between the two distinct populations of striatal projecting neurons, the striatopallidal and the striatonigral MSNs, with the latter population being more consistently involved in LID induction, as suggested by some recent reports [61, 70, 92]. A recent *in vivo* electrophysiological study has given substantial foundation to the distinction between direct and indirect pathways suggesting that a range of different dysfunctional changes in these two populations of projecting neurons may concur to the induction of LID. One interesting aspect that comes out from this paper is that also striatopallidal neurons present specific alterations of synaptic plasticity in response to L-DOPA, although the study leaves open unresolved questions regarding the relevance of these findings for *in vivo* behavior [93].

Besides the distinct contribution of direct and indirect pathways to LID, several lines of evidence support the idea that also striatal regional compartmentalization matters in the response to L-DOPA. Within the striatum, it is possible to distinguish two compartmentalizations, whose activation can be modulated by striatal interneurons: the matrix, including the direct and indirect pathway MSNs that form parts of sensorimotor and associative circuits, and the striosomes, which contain MSNs that receive input from parts of limbic cortex and project directly or indirectly to the dopamine-releasing neurons of the *substantia nigra pars compacta*.

An interesting recent review has strengthened this idea, discussing the role of imbalances between striatal striosome and matrix functions in relation to neurodegenerative disorders, including LID [94]. Findings in support of this idea may have important implications in the perspective of considering PD and LID as network disorders that cause a range of motor and nonmotor symptoms.

## 6. Concluding Remarks

We have discussed seminal and recent papers that explored the mechanisms underlying the establishment of aberrant forms of synaptic plasticity at glutamatergic corticostriatal synapses in LID experimental models. We have also provided an overview of recent studies dealing with novel aspects of the multifaceted L-DOPA effect. Taken together, all the reviewed studies strongly support the notion of a failure of the principal scaling down mechanisms at corticostriatal synapses as a major mechanism in the development of LID. The scenario emerging from these findings is predictive of a more complex pattern of altered plasticity that involves structural and functional changes within the striatal circuitry and opens new perspectives for future electrophysiological investigations.
